# An enzyme-mimicking reactive oxygen species scavenger targeting oxidative stress-inflammation cycle ameliorates IR-AKI by inhibiting pyruvate dehydrogenase kinase 4

**DOI:** 10.7150/thno.101229

**Published:** 2024-11-04

**Authors:** Wenfang He, Chenguang Ding, Ting Lin, Binqi Wang, Wenjing Wang, Zhichao Deng, Taian Jin, Yiwei Shang, Danna Zheng, Ting Bai, Mingzhen Zhang, Runqing Li, Juan Jin, Qiang He

**Affiliations:** 1 Department of Nephrology, the First Affiliated Hospital of Zhejiang Chinese Medical University (Zhejiang Provincial Hospital of Chinese Medicine), Hangzhou, Zhejiang, 310003, China.; 2 Department of Kidney Transplantation, Nephropathy Hospital, The First Affiliated Hospital of Xi'an Jiaotong University, Xi'an, Shaanxi, 710061, China.; 3 Department of Gastroenterology, the Second Affiliated Hospital and Yuying Children's Hospital of Wenzhou Medical University, Wenzhou, Zhejiang, 325024, China.; 4 School of Basic Medical Sciences, Xi'an Jiaotong University, Xi'an, Shaanxi, 710061, China.; 5 Urology & Nephrology Center, Zhejiang Provincial People's Hospital, Affiliated People's Hospital, Hangzhou Medical College, Hangzhou, Zhejiang, 310003, China.; 6 Department of Cardiovascular Medicine, the First Affiliated Hospital, Xi'an Jiaotong University, Xi'an, Shaanxi, 710077, China.; 7 Department of Radiology, the First Affiliated Hospital of Xi'an Jiaotong University, Xi'an, Shaanxi, 710061, China.

**Keywords:** Acute kidney injury, ROS scavenger, Antioxidant, Anti-Inflammatory, Pyruvate dehydrogenase kinase 4

## Abstract

**Rationale**: Ischemia-reperfusion-induced acute kidney injury (IR-AKI), characterized by the abrupt decline in renal function, is distinguished by the intricate interplay between oxidative stress and inflammation. In this study, a reactive oxygen species (ROS) scavenger-CF@PDA was developed to effectively target antioxidant and anti-inflammatory pathways to disrupt the oxidative stress-inflammation cycle in IR-AKI.

**Methods**: UV-vis absorption spectra, FTIR spectra, and TEM were employed to determine the successful construction of CF@P. ABTS, TMB, and NBT analyses were performed to detect the antioxidant ability and enzyme-mimicking ability of CF@P. *In vitro* and *in vitro*, the antioxidant/anti-inflammatory effect of CF@P was detected by MTT, qPCR, fluorescence, and flow cytometry. Multi-omics revealed the mechanism of CF@P in IR-AKI therapy, and molecular docking was further used to determine the mechanism. MRI and photoacoustic imaging were employed to explore the dual-mode imaging capacity of CF@P in IR-AKI management.

**Results**: CF@P could disrupt the oxidative stress-inflammatory cascade by scavenging ROS, reducing pro-inflammatory cytokines, and modulation of macrophage polarization. Subsequent multi-omics indicated that the renal protective effects may be attributed to the inhibition of pyruvate dehydrogenase kinase 4 (PDK4). Metabolomics demonstrated that CF@P could improve the production of antioxidant compounds and reduce nephrotoxicity. Additionally, CF@P exhibited promising capabilities in T1-MRI and photoacoustic imaging for AKI management.

**Conclusions**: Collectively, CF@P, possessing antioxidant/anti-inflammatory properties by inhibiting PDK4, as well as imaging capabilities and superior biocompatibility, holds promise as a therapeutic strategy for IR-AKI.

## Introduction

Acute kidney injury (AKI), an abrupt stoppage of kidney function, is primarily based on increased serum creatinine levels and reduced urinary output and typically lasts for up to a week 1. In a follow-up cohort study in Berlin, a total of 32,338 patients met AKI criteria at least one time point, surprisingly high up to 31.3% of the total population 2. Numerous studies have shown that AKI is developed by a variety of causes, usually mainly by ischemia-reperfusion (IR), sepsis, kidney toxins, or drugs 3. Among these, IR-AKI is the primary reason for AKI acquired in hospitals among patients who have undergone kidney transplants and major surgeries. Besides, IR-AKI is associated with significant short and long-term morbidity and mortality as the major complication of hospitalized patients, especially in critically ill patients 4-6. The significant morbidity and mortality are usually associated with remote multiple organ dysfunction, such as liver dysfunction, cardiorenal syndrome, and brain dysfunction 3. Thus, it is urgent to discover and get effective therapy as soon as possible during the AKI management period.

Currently, there is a lack of highly effective therapy for the treatment of IR-AKI, with supportive treatment and antioxidants such as NAC serving as primary strategies in clinical practice. Consequently, the early identification and treatment of IR-AKI to prevent disease progression has emerged as a key area of focus. It is recognized that oxidative stress and inflammation play a critical role in the pathogenesis of IR-AKI, with these factors mutually exacerbating each other in a detrimental cycle 7, 8. It is well known that ROS overproduction will generate an oxidative stress state. Oxidative stress can increase the production of inflammatory cytokines, and likewise, an increase in inflammatory cytokines can stimulate the production of free radicals 9, 10. In other words, ROS can serve as a cross-linking point between oxidative stress and the inflammatory cycle. Therefore, focusing on the interplay between oxidative stress and inflammation emerges as a promising approach for the management and prevention of IR-AKI 11. This objective can be achieved by implementing interventions that target the reduction of ROS accumulation, modulation of oxidative stress reactions, and suppression of inflammatory cell infiltration.

Numerous studies have shown that nanomaterials, like nanozymes, play an excellent role in inflammation and oxidative stress-related diseases due to their superiority in size-mediated, RONS scavenging, surface-engineered, and biochemical modifications, especially in AKI 12-17. One class of nanomaterials containing natural phenolic compounds, due to their green source, further reduce systemic toxicity and are employed to possess various biological effects such as anti-inflammatory, antioxidant, anti-apoptotic, and immunomodulatory 18-22.

Curcumin (Cur), a representative natural antioxidant and anti-inflammatory, is hailed as a "miracle drug of the future" and a number of clinical trials have shown its safety and feasibility 19, 23-26. Cur can be self-assembled with Fe^3+^ to form the ultra-small nanoparticle- Cur-Fe (CF), which not only improves the solubility of Cur but also helps easy passage through the glomerular filtration membrane and exerts whole-journey anti-inflammatory and antioxidant effects 27. Meanwhile, iron element endows it with excellent magnetic resonance imaging (MRI) imaging function, which can provide additional help for the management of IR-AKI 28-31. It's worth noting that CF has a faster renal clearance and intracellular accumulation due to its ultra-small size.

To prolong the retention time and enhance therapeutic efficacy while avoiding the toxicity caused by the long-term accumulation in the kidneys, we coated CF with a biodegradable antioxidant polydopamine to develop an enzyme-mimicking responsive and degradable ROS scavenger termed CF@PDA (CF@P). Studies have shown that PDA has superoxide dismutase (SOD) enzyme activity and catalase (CAT) enzyme activity, and its related materials show excellent characteristics in clearing ROS, inactivating free radicals, and reducing inflammatory damage in a variety of diseases, such as AKI, cerebral ischemic stroke, and so on 32, 33. The inflammatory microenvironment with high expression of H_2_O_2_ in the kidneys during IR-AKI can promote the gradual degradation of PDA and the release of ultra-small particles of CF through renal tubules, which increases the drug retention time to a certain extent while avoiding the toxicity caused by long-term accumulation. Further, PDA has excellent photoacoustic imaging ability. As an exogenous medium, it can avoid the influence of endogenous hemoglobin and make the imaging clearer and more accurate. Studies have also shown that it can be used for the early diagnosis and evaluation of kidney diseases 34.

**Scheme 1:**
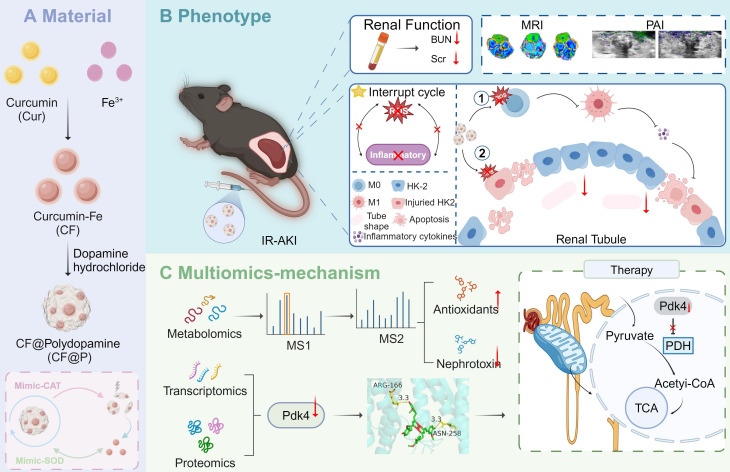
** Schematic illustration of CF@P preparation and potential therapeutic mechanisms in IR-AKI. (A)** The synthesis steps of CF@P and its free radical scavenging ability. **(B)** The protective function of CF@P on kidneys makes efforts by disrupting the oxidative stress-inflammatory cascade. Additionally, CF@P exhibited promising capabilities in T1-MRI and photoacoustic imaging for the management of AKI. ① it represents that CF@P inhibits type 1 macrophage differentiation, reduces the production of pro-inflammatory cytokines, and thereby reduces the harm to the kidney; ② it represents that CF@P can help protect renal tubular epithelial cells from damage caused by oxidative stress and pro-inflammatory cytokines; **(C)** The underlying mechanism of CF@P on IR-AKI. Transcriptome and proteome results showed that CF@P relieved kidney damage in IR-AKI animals by inhibiting Pdk4. Furthermore, metabolomics showed that CF@P increased the production of antioxidant substances and reduced nephrotoxins. This Figure was created by Figdraw and Biorender.

In this work, we successfully constructed an enzyme-mimicking responsive and degradable ROS scavenger termed CF@P for the management of IR-AKI (**Scheme 1**). With systematic administration, CF@P was enriched in the kidneys and exerted renal protective effects to disrupt the oxidative stress-inflammatory cycle through the scavenging of ROS, reduction of pro-inflammatory cytokines, and modulation of macrophage polarization. Subsequent multi-omics results indicated that the renal protective effects may be attributed to the inhibition of PDK4. Metabolomics analysis demonstrated that CF@P could improve the production of antioxidant compounds and reduce nephrotoxicity. Additionally, CF@P exhibited promising capabilities in T1-MRI and photoacoustic imaging for the management of AKI. It is convincing that CF@P holds promise as a therapeutic strategy for IR-AKI.

## Materials and Methods

### Materials

Curcumin, Ferric chloride hexahydrate, and Dopamine hydrochloride were acquired from Aladdin (Shanghai, China). Sigma-Aldrich (St. Louis, MO, USA) supplied the LPS. The total antioxidant capacity assay kit with 2, 2'-azino-bis(3-ethylbenzothiazoline-6-sulfonic acid) (ABTS) method and 2,7-dichlorofluorescein diacetate (DCFH-DA) probe were purchased from Beyotime Biotechnology in Wuhan, China. Dojindo Research Institute of Chemistry (Dojindo, Japan) created the superoxide dismutase assay kit. Promokine in Heidelberg, Germany supplied the fluorescent lipophilic dyes (DiL). MTT, Tunel Cell Apoptosis Detection Kit, and IL-6 antibody were purchased from Servicebio in Wuhan, China, while CD80 antibody was obtained from BD biosciences in San Jose, USA.

### Preparation of CF@P

Firstly, CF was synthesized using the following methods. To summarise, 20 mg of FeCl_3_·6H_2_O was added slowly to the Methanol solution in 1 mL methanol with stirring. Afterward, 10 mg of curcumin were dissolved in 1 mL of methanol, slowly added to the previous solution while subjected to ultrasound, and then stirred for 3 h. The CF solution was acquired, dialyzed in water overnight to eliminate methanol, and refrigerated at 4 °C for further use. A portion of the CF was freeze-dried and weighed to determine its concentration. Next, CF@P was synthesized in the following steps. 9 mL Tris-HCl buffer solution (PH: 8.5, 1 mM) was mixed with 1 mL CF, followed by the addition of the equivalent mass ratio of dopamine hydrochloride (1 : 12). The mixture was stirred constantly for 6 h, followed by centrifugation at a speed of 10,000 rpm for 10 min to gather the sediment that formed. The gathered sediment was rinsed three times with double distilled water to eliminate unattached elements. The obtained substance was CF@P and subsequently kept at 4 °C for further use.

### Characterization of CF and CF@P

The zeta potential and the hydrodynamic diameter (Dh) of CF and CF@P were measured by Malvern Zeta sizer Nano ZS90. The Thermo-Scientific Evolution 201 ultraviolet spectrophotometer was used to measure the UV-vis absorption spectra. In contrast, the Bruker Fourier transformation infrared spectrophotometer from Germany was used to record the FTIR spectra. Furthermore, X-ray photoelectron spectroscopy (XPS, Thermo Fisher ESCALAB Xi +) was utilized to examine the elemental composition and valence states. Transmission electron microscopy (TEM, JEM-2100F) operating at 200 kV was used to analyze the CF and CF@P.

### ROS Scavenging Ability Test

Commercial test kits were employed to compare the total antioxidant capacity and SOD enzyme activity of CF and CF@P. Moreover, we performed them according to instructions.

TMB assays: Fenton's reagent (Fe^2+^: 10 μM; H_2_O_2_: 50 μM) and TMB (0.3 mM) were mixed and then incubated with CF and CF@P (2.5, 5, 10, 15, and 20 μg/mL), respectively. After the reaction, oxTMB was monitored by a UV spectrophotometer.

### Cell Culture and Test

HK-2 and RAW 264.7 cells were grown in a specific medium from Procell (Wuhan, China) at 37 °C and 5% CO_2_.

Uptake experiments were performed on RAW 264.7 cells and HK-2 cells, which were treated with DiL - CF@P for 2, 4, 6, and 8 h. After staining with cytoskeleton markers, the cells were fixed with 4% paraformaldehyde and rinsed multiple times with PBS. DAPI staining was performed and rewashed several times. The results were obtained using an upright microscope system and flow cytometry.

RAW 264.7 and HK-2 cells were seeded in 6-well plates at a suitable density for intracellular examinations of antioxidants and anti-inflammatory characteristics. Subsequently, the cells were co-incubated with CF@P (10, 20, 40 μg/mL) for 6 h, followed by washing, and then co-cultured with H_2_O_2_ (600 μM) for another 6 h. 2, 7-dichlorofluorescein diacetate (DCFH - DA, 20 μM) and dihydroethidium (DHE, 1 μM) were utilized to quantify the amounts of intracellular ROS. RAW 264.7 cells and HK-2 were placed in 96-well plates and exposed to CF@P (10, 20, 40 μg/mL) and H_2_O_2_ (600 μM) for 6 h, respectively. Subsequent MTT assays were conducted to assess cell viability and evaluate the antioxidant properties of CF@P.

To conduct the macrophage polarization test, RAW 264.7 cells were treated with CF@P at 10, 20, and 40 μg/mL concentrations for 6 h, followed by overnight incubation with 1 μg/mL lipopolysaccharide (LPS). The polarization of macrophages was assessed by staining RAW 264.7 cells with CD 80 markers and performing a flow cytometry assay.

### Real Time-PCR

RAW 264.7 and HK-2 cells were treated with CF@P (10, 20, 40 μg/mL) and LPS (1 μg/mL, 10 mg/mL, respectively) and RNA was obtained by a commercial kit from Beyotime Biotechnology following co-incubation. The kit was also suitable for animal kidney tissue. And The qPCR ran as usual. Suitable primer sequences are listed in Table S1.

### Animal Experiments

C57BL/6 male mice aged 6-8 weeks were obtained from GemPharmatech Co. in Nanjing, Jiangsu Province, China. The animals were housed at a temperature of 22-25 °C, with humidity maintained at 65±5%, exposed to a 12 h light-dark cycle, and provided with regular access to drinking water. Every examination followed the regulations established by the Institutional Animal Care and Use Committee at Xi'an Jiaotong University.

Briefly, the surgery steps and therapy of the IR-AKI model were as follows: 1) Clamped both renal pedicles with hemostatic clips; 2) After 35 min, released the bilateral hemostatic clamps; 3) Within 10 min after releasing the hemostatic clamp, the treatment group and the IR-AKI group were injected with CF@P (2.5 mg/Kg) and normal saline, respectively. Mice were euthanized based on the designated time frame, and their kidneys were harvested for H&E staining, PAS staining, ROS analysis, qPCR, and immunostaining.

### Western Blotting

Western blotting was performed using the following antibodies: PDK4 Polyclonal antibody (1:2000, Proteintech, China), Alpha Actinin Polyclonal antibody (1:10000, Proteintech, China) and HRP-linked goat anti-rabbit/mouse IgG (1:10000, Proteintech, China).

### Immunostaining

The kidney tissues were carried out into frozen sections. After washing with PBS 3 times, IL-6 antibody, DCFH-DA, and DHE probes were incubated with the above-frozen sections following the instructions.

### Evaluation Method of Renal Tubular Injury

The Paller scoring method was used to score the degree of renal tubular injury 35. Scoring criteria: 1 point for obvious dilation of renal tubules, 1 point for a brush-like edge injury, and 2 points for detachment; the presence of detached or necrotic cells in the renal tubular lumen was 1 point, the tubular type was 2 points, and the total score was 5 points.

### Multi-omics Processing of Animal Kidney Tissue

The kidney tissues of each group were processed and analyzed with the assistance of LC-BIO (Hangzhou, China). In the transcriptome analysis, differentially expressed genes were identified based on a foldchange ≥ 2 (equivalent to an absolute log_2_ FC ≥ 1) and a q value <0.05 (where q is the corrected *p-*value) (|log_2_ FC| ≥ 1 & q < 0.05). In proteomics, the standards for identifying differentially expressed proteins include a *p-*value less than 0.05, and a foldchange ≤ 0.67 or foldchange ≥ 1.5(Chi-square test or Student's t-expression test). In the same way, for metabonomics, XCMS software was employed for peak extraction and quality assurance in metabonomics, while metaX software was utilized for identifying metabolites. The Chi-square test or Student's t-expression test was utilized to validate the distinct metabolites, satisfying the criterion of FC ≥ 1.2 or FC ≥ 1, *p*-value < 0.05, and VIP ≥ 1. The set of genes from the mitochondria of mice was obtained from the MitoCarta 3.0 database 36.

### Molecular Docking

Autodock Vina 1.2.2 was utilized to examine the connections and binding affinities between CF and PDK4. The molecular structure of CF was drawn as previous studies showed 37. The 3D coordinates of PDK4 (O70571) were downloaded from the Uniprot (https://www.uniprot.org). Before conducting docking analysis, the protein and molecular files were converted to PDBQT format, with water molecules removed and polar hydrogen atoms included. The grid box was positioned at the center to encompass the range of each protein and allow for unrestricted molecular motion. The grid was configured to be angstroms by 30 angstroms by 30 angstroms, with a spacing of 0.05 nm between grid points. Molecular docking analysis was performed using Autodock Vina 1.2.2 (http://autodock.scripps.edu/) and visualized by Pymol.

### MRI and Photoacoustic Imaging of CF@P

For MRI, different concentrations of CF@P were prepared and the material was subjected to T_1_-weighted imaging in MINIMR60. Similarly, after the injection of CF@P into two groups of mice under respiring anesthesia, the detection was performed under the instrument at different time points.

For photoacoustic imaging, Fujifilm Visual Sonics Vevo® LAZR-X was employed to detect and record. Firstly, the wavelength of CF@P was detected by the instrument, and 695 nm was determined. Secondly, the two groups of mice were subjected to testing after CF@P injection at different time points. And it should be noted that mice need to be depilated.

### Statistical Analysis

GraphPad Prism 8.0 software was used to conduct statistical analysis on all the outcomes. A Student's t-test was employed to compare the two groups. The results were presented as mean ± standard deviation (SD). A statistically significant difference was observed with a significance level of *P* < 0.05.

## Results and Discussion

### Synthesis and Characterization of CF and CF@P

**Figure 1:**
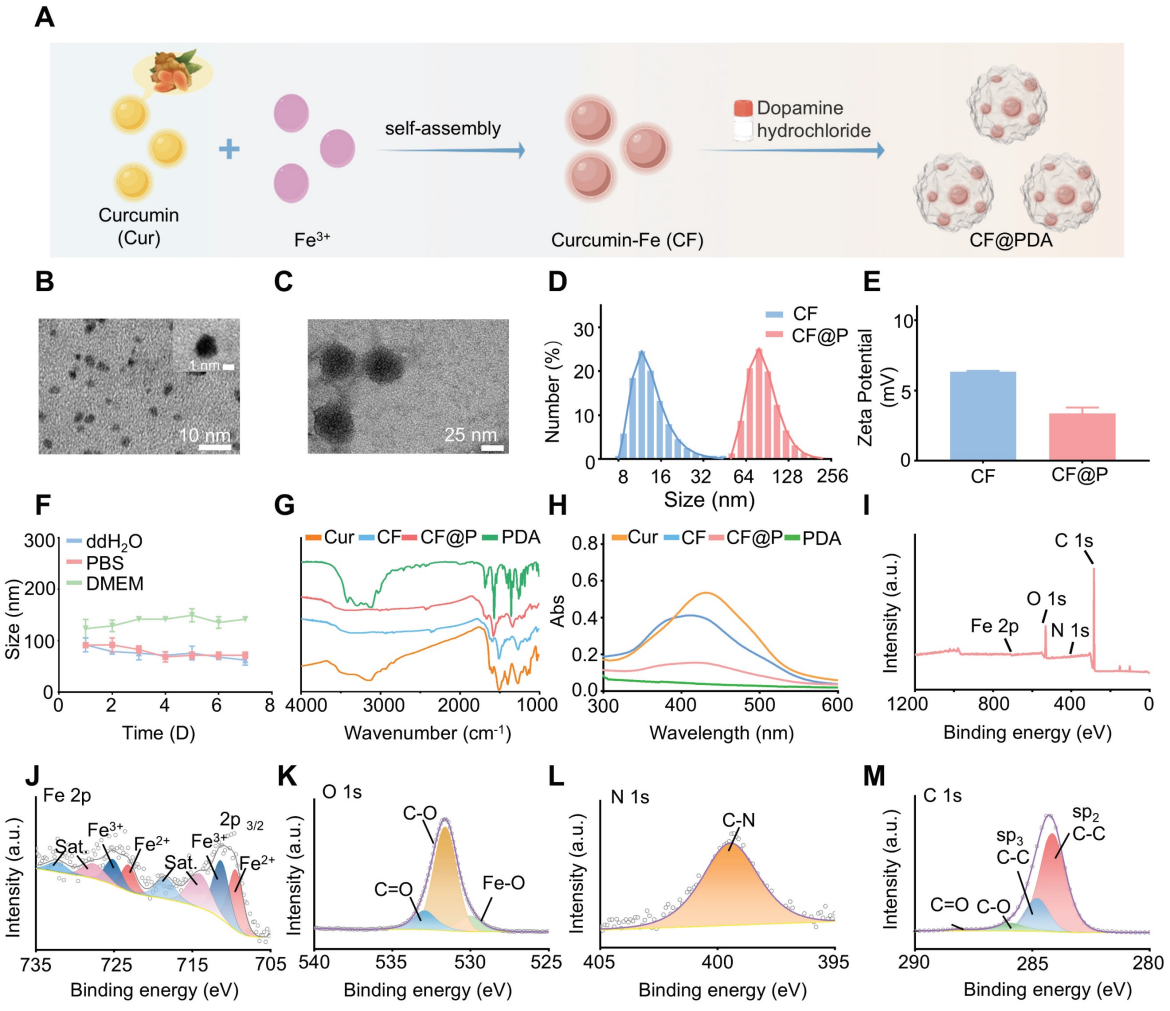
** Synthesis and characterization of CF and CF@P. (A)** Synthesis steps of CF and CF@P. This Figure was created by Figdraw. **(B)** TEM image of CF (scale bar, 10 nm, 1nm, respectively).** (C)** TEM image of CF@P (scale bar, 25 nm).** (D)** Representative hydrodynamic diameters of CF@P and CF. **(E)** Zeta potential of CF@P and CF. **(F)** Stability of hydrodynamic diameters of CF@P over 7 days in different systems. FTIR spectra** (G)** and UV-vis spectra **(H)** of Cur, CF, PDA, and CF@P.** (I)** Wide-range XPS patterns of CF@P. Fe 2p **(J)**, O1 s **(K)**, N1 s **(L)**, and C1 s **(M)**. Data are presented as mean ± SD (N = 3).

The specific synthesis steps of the CF@P are detailed in **Figure 1A**. Initially, CF was produced through the self-assembly of Curcumin and Fe^3+^, with the transmission electron microscope (TEM) revealing an approximate size of 2-3 nm (**Figure 1B and Figure 1D**). CF was then coated with PDA, and TEM showed CF@P particles to be roughly 40-50 nm in size (**Figure 1C-D**). The zeta potential measurements indicated that both CF and CF@P carried positive charges (6.33±0.07 mV and 3.37±0.43 mV, respectively), enhancing their ability to cross the glomerular filtration membrane (**Figure 1E**). Additionally, the stability of the 7-day particle size in various systems confirmed the high stability of CF@P (**Figure 1F**). The FTIR spectra of CF and CF@P showed decreased infrared intensity at 1150-1200 cm^-1^ (HO-C stretching band), indicating the connection between Fe^3+^ and HO-C components of curcumin. Characteristic absorption peaks at 1610-1510 cm^-1^ in PDA indicated the stretching vibration absorption peaks of the benzene ring 38, 39 (**Figure 1G**). UV-visible spectroscopy analysis showed a distinctive absorption peak around 412 nm for CF, indicating the successful combination of Cur and Fe^3+^ (**Figure 1H**). CF@P also exhibited a characteristic absorption peak at 418 nm. Besides, CF@P had absorption at the range of 300-600 nm, as well as PDA. The chemical bonds of CF@P were examined by HR-XPS spectra (**Figure 1I**). **Figure 1J-M** displayed spectra of Fe 2p, O 1s, N 1s, and C 1s. The HR-Fe 2p spectra confirmed the existence of Fe^2+^ and Fe^3+^ in CF@P. Additionally, the presence of metal oxides (Fe-O), along with C-O and C=O bonds in CF@P was highlighted in the HR-O 1s spectra. Peaks corresponding to C-N were observed in the HR-N 1s spectrum, while the HR-C 1s spectrum displayed peaks assigned to C-C, C-O, and C=O bonds. We employed inductively coupled plasma (ICP) to detect the actual Fe content in CF@P. The results showed that the content was 0.047±0.0004% g/g. These results indicated that CF@P was successfully synthesized.

### Antioxidant Capacity and Enzyme-like Activity of CF@P

**Figure 2:**
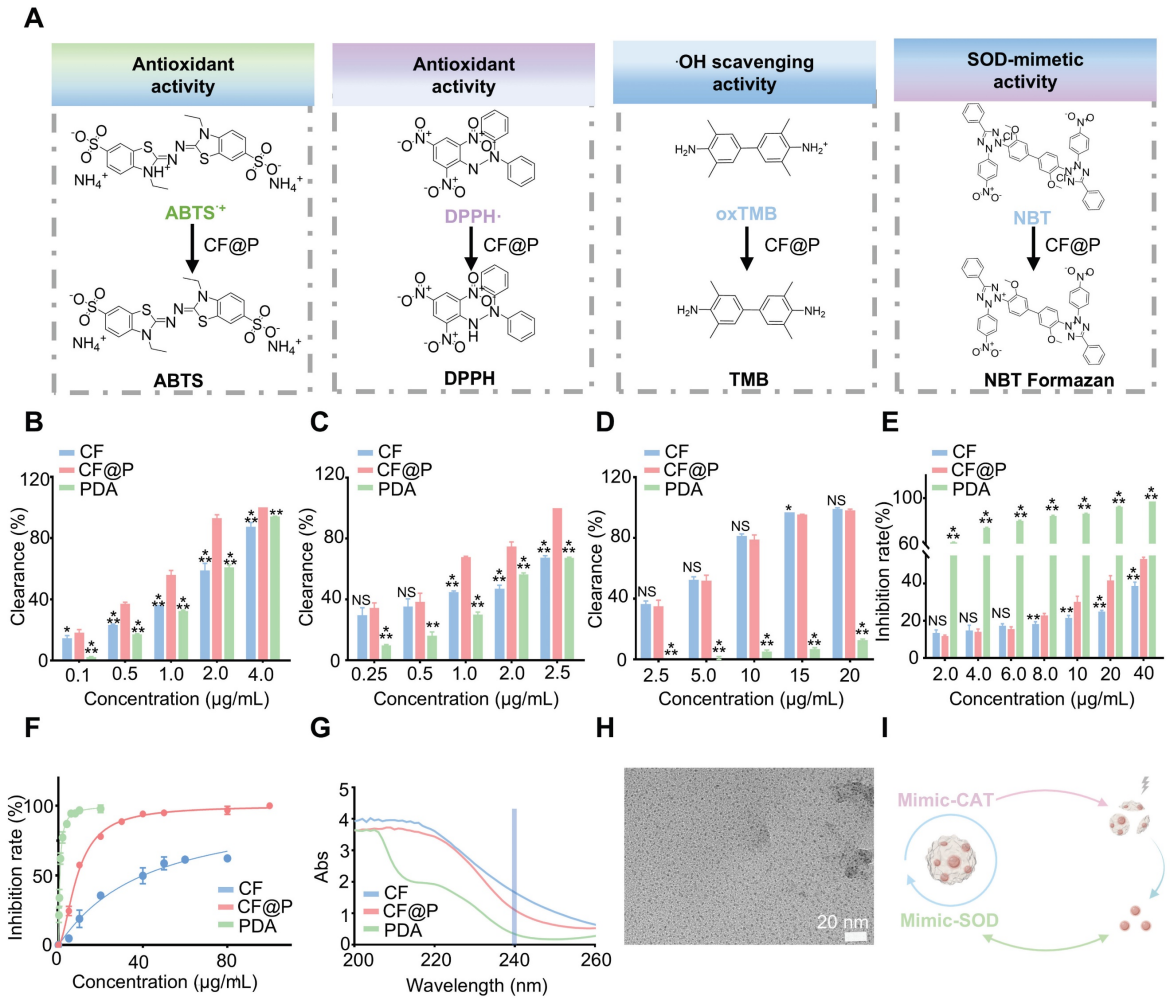
** Antioxidant capacity of CF@P. (A)** The schematic illustrates the detection of the ROS scavenging ability of CF@P. **(B)** Quantitative analysis of ABTS^•+^ scavenging rate by CF@P, CF and PDA at different concentrations (0.1, 0.5, 1.0, 2.0, and 4.0 μg/mL). **(C)** Quantitative analysis of DPPH• scavenging rate by CF@P, CF, and PDA at different concentrations (0.25, 0.5, 1.0, 2.0, and 2.5 μg/mL). **(D)** Quantitative analysis of oxTMB scavenging rate by CF@P, CF, and PDA at different concentrations (2.5, 5, 10, 15, and 20 μg/mL).** (E)** O_2_^•**-**^-scavenging ability of CF@P, CF, and PDA detected by NBT (2, 4, 6, 8, 10, 20, and 40 μg/mL).** (F)** Percentage of superoxide radical elimination catalyzed by SOD-like activity of CF@P, CF, and PDA.** (G)** CAT activity of PDA. **(H)** Representative TEM image of CF@P after incubation with H_2_O_2_. Scale bar, 20 nm. **(I)** Schematic diagram of mimic-enzyme activity of CF@P. This Figure was created by Figdraw. Data are presented as mean ± SD (*P < 0.05, **P < 0.01, ***P < 0.001).

During the progression of IR-AKI and its associated malignant complications, the reperfusion elicits a robust traumatic response associated with increased oxidative stress that damages the kidney 40. A primary goal of using CF@P is to mitigate oxidative stress and neutralize radicals. To confirm the effectiveness of CF@P in eliminating radicals, we analyzed total antioxidant capacity and the elimination of two common ROS: superoxide radicals and hydroxyl radicals (**Figure 2A**).

To determine the comprehensive antioxidant capacity of CF@P, the ABTS^•+^ assay was employed (**Figure 2B**). Under stable conditions, ABTS^•+^ appears as a blue-green color with a maximum absorption peak at 734 nm, which diminishes upon the introduction of antioxidants. Through the ABTS assay, we examined the total antioxidant capacity of CF@P, CF, and PDA. The results showed their excellent antioxidant capacity. Notably, CF@P exhibited a clearance rate of nearly 90% when added at a concentration of 2 μg/mL. Meanwhile, we compared the antioxidant capacity in different solvent systems (anhydrous ethanol) to test the stability of its antioxidant capacity. Nearly 100% DPPH^•^ were oxidized by adding 2.5 μg/mL CF@P, indicating the relatively stable powerful antioxidant capacity of CF@P (**Figure 2C**). 3, 3, 5, 5-tetramethylbenzidine (TMB) was used as an indicator to evaluate the •OH scavenging ability of CF, PDA, and CF@P. The clearance of ox-TMB indicated that both CF and CF@P own excellent •OH scavenging ability and almost had no difference (**Figure 2D, Figure S1A-B**). This result seemed to indicate that CF contributes more to the excellent ability of •OH scavenging ability in CF@P. To assess the O_2_^•**-**^-scavenging ability, we determined it by inhibiting the reduction of nitrogen blue tetrazole (NBT) under light, preliminarily. We could see that there was seldom any difference in the reduction of NBT between CF and CF@P NPs at low concentrations. At a concentration of 8 μg/mL, CF@P showed a notable benefit. And PDA demonstrated significant advantages in this regard (**Figure 2E**).

Superoxide dismutase (SOD) is ubiquitously distributed among animals and plants, functioning as an enzyme responsible for the elimination of superoxide anion free radicals. Commercial Superoxide dismutase (SOD) kits were employed to explore the SOD enzyme activity. The results showed that PDA had higher SOD enzyme activity compared to CF, which might be the reason why the overall SOD enzyme activity of CF@P is superior to CF (**Figure 2F**). Since PDA has CAT enzyme activity, we further tested it by incubating it with H_2_O_2_ and measuring its consumption at 240 nm by UV. **Figure 2G** demonstrates that Abs significantly decreased after incubation with PDA, indicating its CAT activity. However, considering that CF and CF@P themselves have high absorbance at 240 nm, which could easily have a significant impact on the results, we indirectly verified whether CF@P inherits this characteristic of PDA. We, directly through TEM, observed that the PDA degraded and retained the CF after incubation with 600 μM H_2_O_2_ (**Figure 2G**). Overall, the PDA coat significantly improved the antioxidant performance, indicating that CF@P may serve as a promising therapeutic agent for safeguarding against oxidative damage induced by ROS in the context of ischemia-reperfusion acute kidney injury (**Figure 2I**).

### Antioxidant and Anti-inflammatory Effects of CF@P in RAW 264.7 and HK-2 Cells

Macrophages, a significant factor in the pathogenesis of kidney inflammation, are present throughout various stages of disease progression, including inflammation, repair, and fibrosis. Through single-cell sequencing, it was determined that macrophages constitute the predominant immune cell population in diverse AKI models 35. Consequently, we conducted experiments to assess the antioxidant and anti-inflammatory properties of CF@P in Raw264.7 cells under conditions that mimic oxidative stress and inflammation.

**Figure 3:**
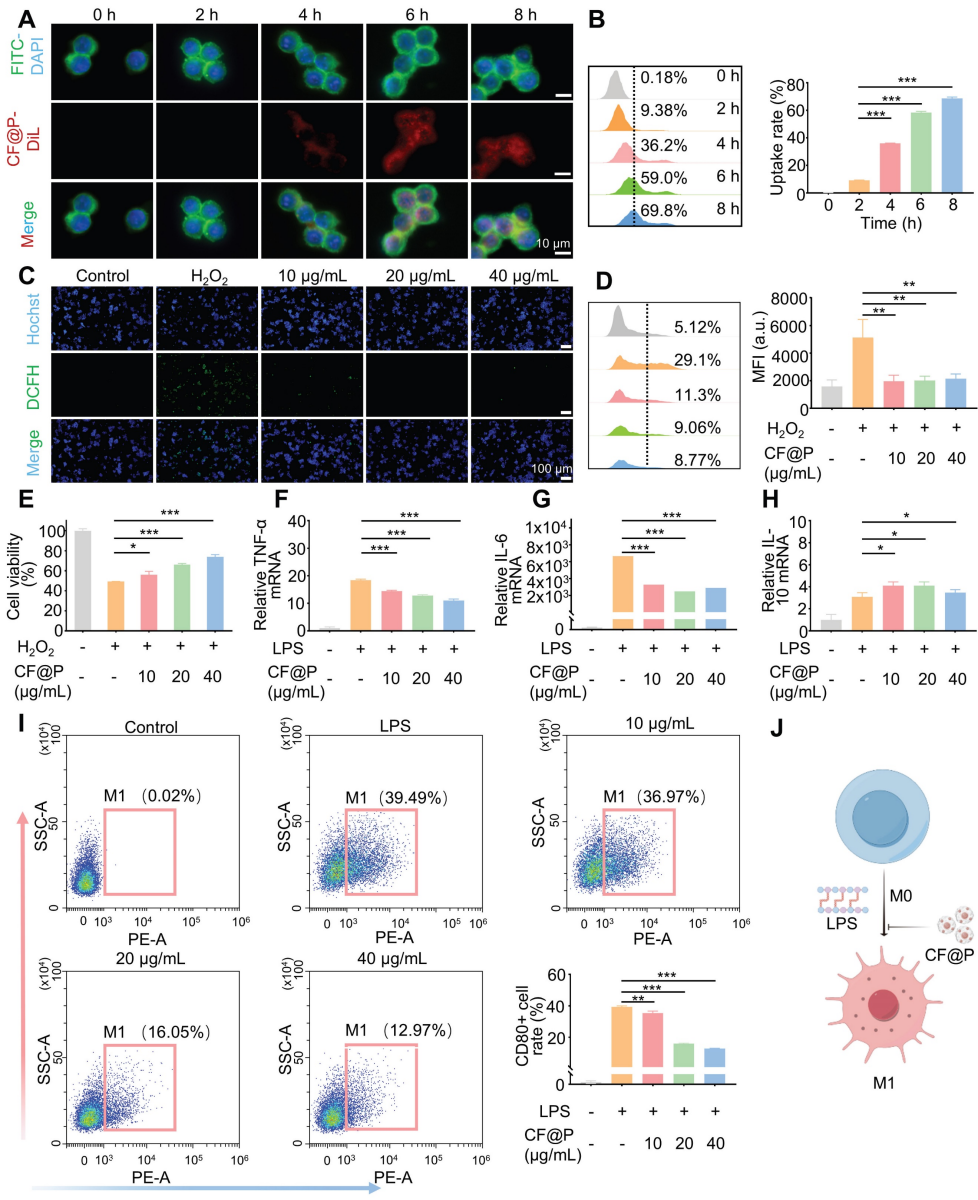
** The cellular internalization and antioxidant/anti-inflammatory effects of CF@P in RAW 264.7 cells. (A-B)** Typical fluorescence images (Scale bar, 10 µm) and flow cytometry of cellular uptake of CF@P in RAW 264.7. **(C-D)** Typical fluorescence images (Scale bar, 100 µm) and flow cytometry of RAW 264.7 cells stained with DCFH-DA. **(E)** Relative viabilities of RAW 264.7 cells with different treatments, including H_2_O_2_ (600 μM) and CF@P (10, 20, 40 μg/mL) with H_2_O_2_. **(F-H)** The mRNA expression levels of TNF-α, IL-6, and IL-10 in RAW 264.7. **(I)** Flow cytometric examination of the M1-phenotype macrophages in different groups.** (J)** Schematic illustration shows the regulation of macrophage phenotype by CF@P and this Figure was created using Figdraw. Data are presented as mean ± SD (N = 3; *P < 0.05, **P < 0.01, ***P < 0.001).

After incubation with DiL-CF@P for different periods in RAW 264.7, 6 h was determined as the suitable incubation time based on fluorescence and flow cytometry results (**Figure 3A-B**). Various levels of CF@P were examined for their ability to remove intracellular ROS using a DCFH-DA probe. The results in **Figure 3C** showed that the group treated with H_2_O_2_ exhibited intense green DCF fluorescence compared to the control group, indicating effective stimulation of ROS generation by H_2_O_2_. The DCF fluorescence intensity decreased significantly after cells were pre-incubated with CF@P. Similarly, flow cytometry revealed that different concentrations of CF@P demonstrated good scavenging ability for ROS (**Figure 3D**). Prolonged exposure to H_2_O_2_ could even induce apoptosis. The MTT analysis results showed that the damage caused by H_2_O_2_ could be effectively inhibited by CF@P, and this result was dependent on the concentration gradient (**Figure 3E**).

In response to injury, macrophages become activated based on specific signals from the damaged microenvironment. And activated macrophages (M1) induced by LPS, which exhibit pro-inflammatory phenotypes, the TNF-α and IL-6 mRNA expression levels were significantly decreased after CF@P pre-incubation (**Figure 3F-G**). Further, IL-10, a recognized anti-inflammatory factor, was significantly elevated in the CF@P treatment group (**Figure 3H**). Meanwhile, reducing the activation of M1 would also be an effective strategy to reduce inflammatory response. We detected the number of M1 in each group by flow cytometry and found that the number of M1 cells was the lowest in the 40 μg/mL CF@P group (**Figure 3I**). Therefore, we believe that CF@P can effectively prevent the activation of M1 induced by LPS (**Figure 3J**). Some studies showed that macrophages could interact with parenchymal cells and have cell communication effects 41, 42. Therefore, reducing the activation of M1 and the production of inflammatory factors will also reduce the indirect damage to parenchymal cells.

**Figure 4:**
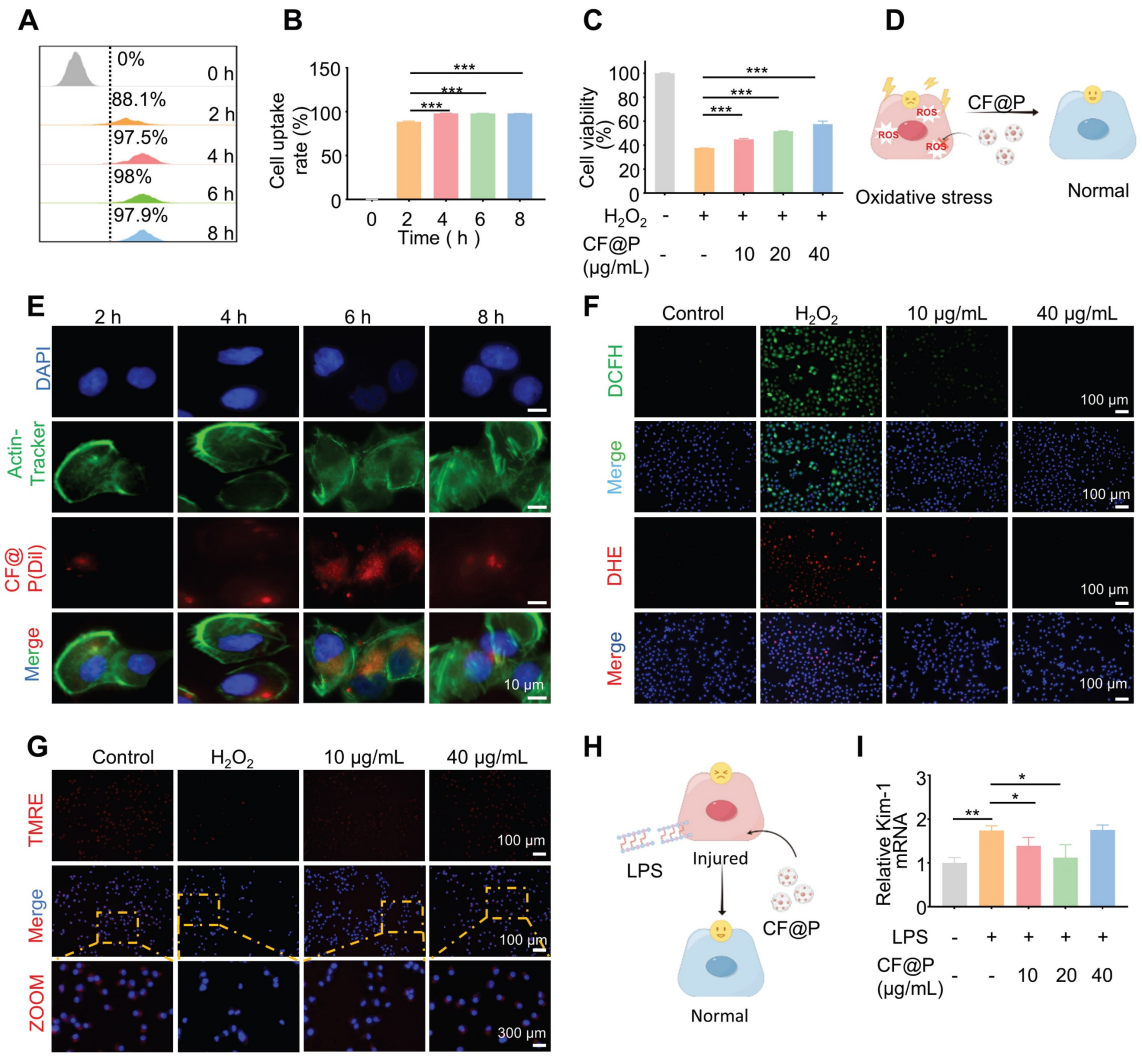
** The cellular internalization and antioxidant/anti-inflammatory effects of CF@P in HK-2 cells. (A)** Relative viabilities of HK-2 cells with different treatments including H_2_O_2_ (600 μM) and CF@P (10, 20, 40 μg/mL) with H_2_O_2_.** (B)** Schematic illustration shows the antioxidant of CF@P in avoiding injury and apoptosis of HK-2. This Figure was created by Figdraw. **(C-E)** Flow cytometry and typical fluorescence images (scale bar, 10 µm) of cellular uptake of CF@P in HK-2. **(F)** Typical fluorescence images of HK-2 cells stained with DCFH-DA and DHE (scale bar, 100 µm). **(G)** Typical fluorescence images of HK-2 cells stained with TMRE (scale bar, 100 µm, 300 µm). **(H)** Schematic illustration shows the anti-inflammatory ability of CF@P to avoid the injury of HK-2 cells. This Figure was created by Figdraw.** (I)** The mRNA level of Kim-1 in HK-2 cells. Data are presented as mean ± SD (N = 3; *P < 0.05, **P < 0.01, ***P < 0.001).

Renal tubular epithelial cells, one of the high-energy metabolic cells, also take important part in the progression of IR-AKI. To further explore the effect of CF@P on renal parenchymal cells, HK-2 cells were employed. After incubation with DiL-CF@P for different periods in HK-2 cells, 6 h was selected as the suitable time for incubation by fluorescence and flow cytometry (**Figure 4A-B, and Figure 4E**). The excessive ROS caused by ischemia-reperfusion may lead to cell apoptosis. However, the MTT analysis results showed that the devastating damage of H_2_O_2_ to HK-2 cells could be effectively eliminated by CF@P and it was concentration gradient dependent (**Figure 4C-D**). Additionally, the DCFH-DA and DHE probes were utilized to study the ability of CF@P to scavenge intracellular ROS. In **Figure 4F**, **Figure S2A-B, and D-E**, the fluorescence intensity of DCF and DHE significantly attenuated in CF@P groups, especially at 40 μg/mL. The scavenging of excess ROS production could help eliminate lipid peroxidation, protein damage, and DNA breakage caused by oxidative stress. The stability of mitochondrial membrane potential (MMP) is conducive to maintaining the normal physiological function of cells. MMP could decline before the early pathological changes of apoptosis and TMRE accumulation decreased, thus red fluorescence decreased.

This phenomenon can be perfectly observed in** Figure 4G, Figure S2C, and Figure S2F**. The H_2_O_2_-induced group showed the MMP was significantly depolarized and red fluorescence decreased after binding with TMRE. This phenomenon was completely reversed in the CF@P group. Besides, the level of kidney injury molecule-1 (Kim-1) mRNA, a recognized marker for tubular injury during AKI, was detected t to ensure the anti-inflammatory of CF@P in HK-2. The results indirectly indicated that CF@P has an anti-inflammatory effect (**Figure 4H-I**). However, high-dose CF@P couldn't resist LPS. This result is interesting. We speculated that it might be the reason that HK2 cells are more sensitive to solute concentration. However, it needed further research to clarify. In conclusion, the results provided compelling evidence that CF@P exhibits robust antioxidant properties, mitigates oxidative stress-induced damage, and suppresses inflammatory responses in RAW 264.7 and HK-2 cells. These findings underscored the therapeutic promise of CF@P in disrupting the interplay between oxidative stress and inflammation in the context of ischemia-reperfusion acute kidney injury *in vitro*.

### MRI Imaging and Photoacoustic Imaging Capabilities of CF@P

**Figure 5:**
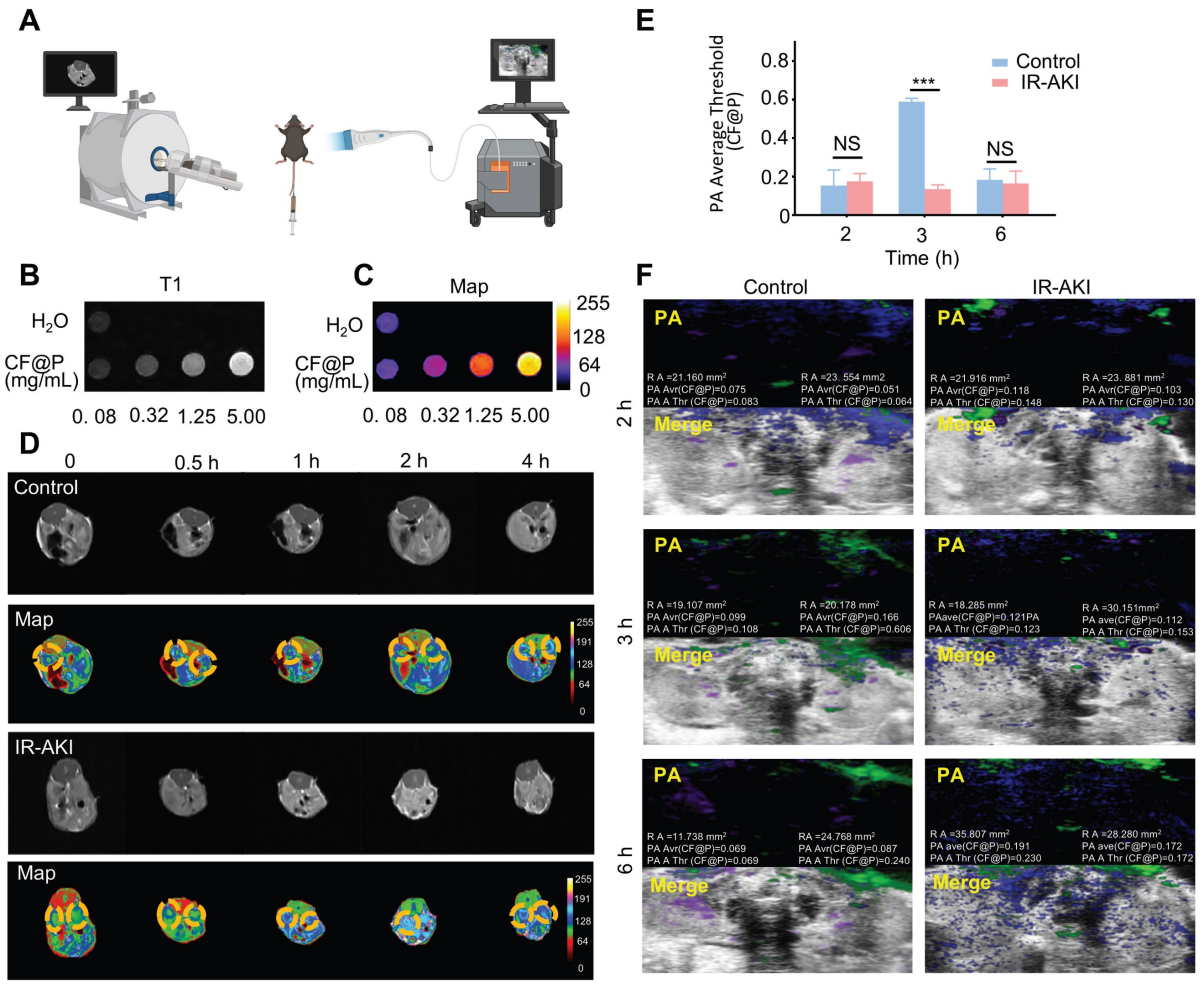
** Dual-mode imaging capability of CF@P in IR-AKI. (A)** Illustration of two model imaging. **(B)** Signal strength picture of CF@P with different mass concentrations under T1-MRI.** (C)** Pseudo-colour graph of Figure (B).** (D)** T1-MRI images and corresponding pseudo-colour graphs of mice at different time points (0, 0.5, 1, 2, 4 h) after CF@P injection in the control and IR-AKI group.** (E-F)** Statistic analysis and Photoacoustic images of kidneys at different time points (2, 3, 6 h) after CF@P injection in control and IR-AKI group. The green represents CF@P, and the purple and blue represent arterial and venous blood, respectively. Data are presented as mean ± SD (*P < 0.05, **P < 0.01, ***P < 0.001).

Due to the high prevalence of AKI, as well as its associated short- and long-term morbidity and mortality rates, the utilization of imaging modalities is imperative for the diagnosis and monitoring of disease progression in patients undergoing AKI management. MRI presents a valuable opportunity for the continuous and non-invasive observation of the development and advancement of AKI towards chronic injury 43. Iron-based nanozymes are recognized as a subset of inorganic nanoparticles that are currently employed in clinical practices for disease diagnosis and therapy 44. Leveraging the biocatalytic properties and physicochemical attributes of iron-based nanozymes, they have found extensive application in various domains such as disease management, immune profiling, antimicrobial activities, cellular monitoring, and other related areas 45-47. PA imaging has demonstrated high effectiveness in detecting kidney diseases, attributed to its enhanced imaging depth and spatial resolution achieved through the integration of optical and ultrasound technologies. Therefore, we hypothesized that CF@P, a nanomaterial known for its superior performance in treating IR-AKI, could also serve a significant role in imaging for AKI management, given its PDA coat and iron element (**Figure 5A**).

*In vivo*, CF@P showed excellent imaging capability under the T1 mapping (**Figure 5B**). Moreover, the images of increasing concentration were more intuitive in the pseudo-colour setting (**Figure 5C**). In a 4 h observation about the two groups of mice injected with CF@P, we found that kidney images were more pronounced and cleared in the IR-AKI group, especially at 2 h (**Figure 5D**). These results suggested that CF@P had renal enrichment and* in vivo* imaging capabilities. Further, we speculated that the apparent contrast may be due to the degradation of the PDA coat under oxidative stress, and thus more internal CF flowed into the kidney, which made the kidneys in the IR-AKI group more explicit.

Meanwhile, the photoacoustic imaging capability of CF@P was also demonstrated. Similarly, we compared CF@P images in the normal and IR-AKI groups over 6 h. After deleting the background signal intensity of 0 h, the photoacoustic images and statistical results were shown in **Figure 5E-F**. Green represents CF@P, and purple and blue represent arterial and venous blood, respectively. After the injection of CF@P, the PA Average Threshold (CF@P) reached the highest value of 0.606 in the normal group at 3 h. This threshold value was higher than the highest threshold in the IR-AKI group due to the degradation of PDA under the inflammatory microenvironment with high expression of H_2_O_2_. To some extent, it suggested that a decrease in the highest threshold of CF@P might be used to detect the occurrence of IR-AKI. The value of CF@P average in renal remained relatively stable and higher than the normal group in the IR-AKI group, which could be related to the slow blood flow during IR-AKI. In summary, during the period of IR-AKI management, the dual-mode imaging ability of CF@P could provide practical assistance for IR-AKI identification, and it was believed to have the potential to be used in the clinic.

### Therapeutic Effects of CF@P against IR-AKI *in Vivo*

**Figure 6:**
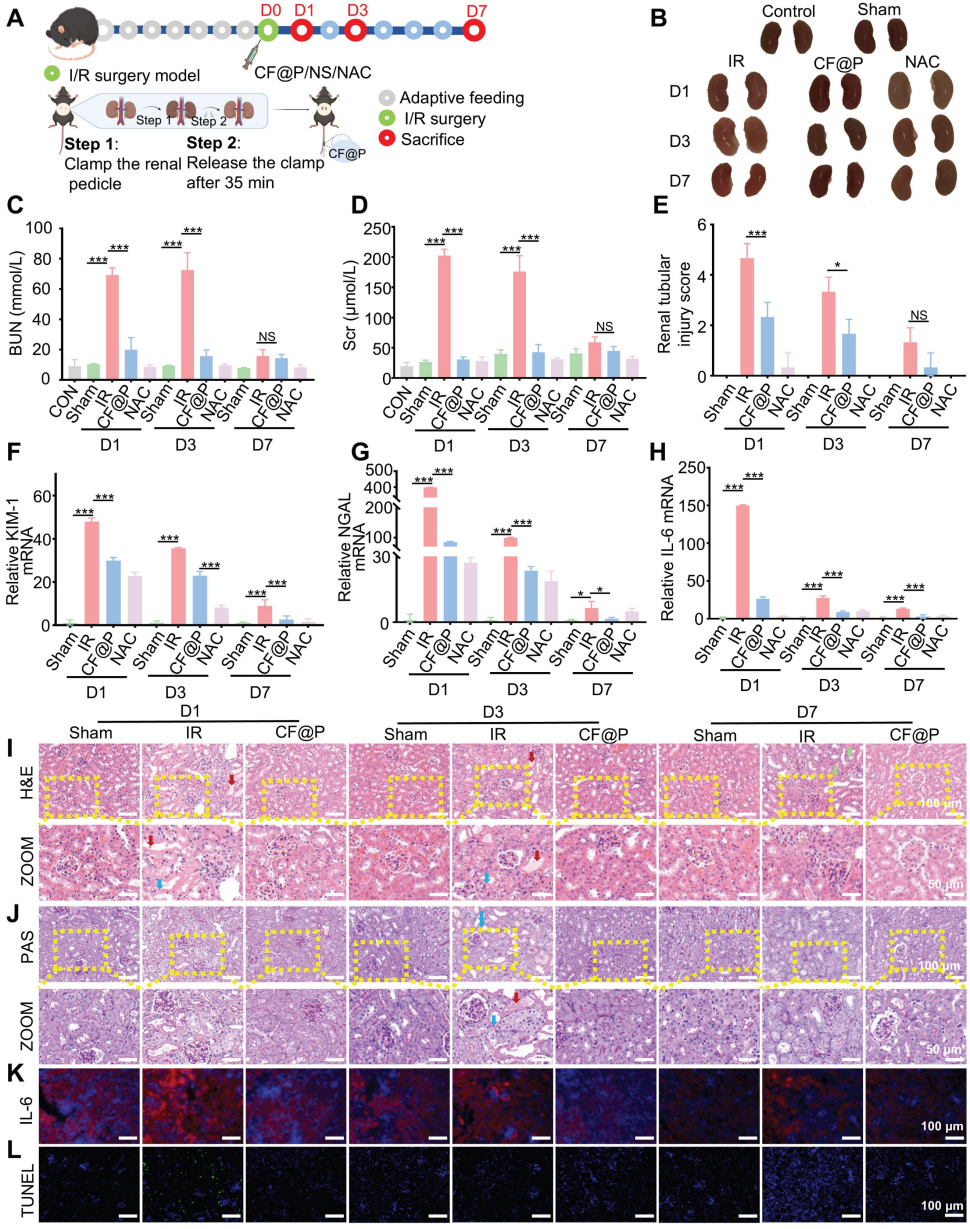
** Therapeutic efficacy of CF@P against IR-AKI. (A)** Schematic showing the experimental procedure for the treatment of IR-AKI mice and the steps of the IR-AKI model. This Figure was created by Figdraw. **(B)** Representative photos of the kidney in different groups. The levels of BUN **(C)** and Scr** (D)** in groups (n = 6).** (E)** Analysis of renal tubular injury score for H&E photos of groups (n = 3). **(F-H)** The mRNA expression levels of Kim-1, NGAL, and IL-6 in groups (n = 3). Typical H&E** (I)**, PAS **(J)**, and ZOOM images of kidney tissues in different groups (Red arrow: shed epithelial cells, cell debris, and casts in the renal tubule lumen; Blue arrow: exposed renal tubular basement membrane; Green arrow: renal interstitial fibrosis. scale bar, 100 µm, and 50 µm, respectively). **(K)** Typical immunofluorescence images of IL-6 in groups (scale bar, 100 µm). **(L)** Typical fluorescence images of Tunel in groups (scale bar, 100 µm). Data are presented as mean ± SD (*P < 0.05, **P < 0.01, ***P < 0.001).

A mouse model of IR-AKI was employed to investigate the therapeutic efficacy of the CF@P *in vivo*, and a series of parameters were used. Groups were formed randomly from C57BL/6 mice, including normal control, D1-Sham, D1-IR, D1-CF@P, D1-NAC, D3-Sham, D3-IR, D3-CF@P, D3-NAC, D7-Sham, D7-IR, D7-CF@P, D7-NAC. Following a week of acclimation, the mice in each group underwent a bilateral renal ischemia-reperfusion injury procedure lasting 35 min, except the blank control and sham groups. Then, a 2.5 mg/Kg dose of CF@P or NAC was injected through the tail vein within 10 min of the completion of the surgery. At the same time, the control group and sham group received an injection of an equal volume of saline solution. The mice were sacrificed at 1, 3 and 7 days after surgery, as** Figure 6A** was shown.

The IR group's body weight decreased significantly and steadily increased around 4 days after surgery. Surprisingly, the body weight of the CF@P group and NAC group began to increase early on the second day after surgery (**Figure S3A**). The isolated kidneys were reddish brown in the control and sham groups, undoubtedly. However, the colour in the IR-AKI group was paler than other therapy groups, and this difference was gradually restored at D7. Compared with the IR-AKI group, CF@P injection significantly improved the renal appearance colour of IR-AKI (**Figure 6B**). In contrast, after CF@P treatment, the mice display significantly low BUN and Scr levels, similar to those in the positive group (NAC), indicating that CF@P can effectively improve the damaged renal function of AKI mice (**Figure 6C-D**). The low levels of BUN and Scr in the D1-CF@P and D3-CF@P treatment groups indicated that it could effectively shorten the duration of the disease and help avoid further development.

Additionally, KIM-1 and neutrophil gelatinase-associated lipocalin (NGAL), recognized markers of acute kidney injury, were used to evaluate the therapeutic effect of CF@P by qPCR. The results showed that KIM-1 and NGAL mRNA were highly expressed in all IR groups, and CF@P significantly decreased the expression of these two factors and closed to the positive groups (**Figure 6F-G**). The therapeutic effect of CF@P on IR-AKI can also be directly observed from HE and PAS detection. In H&E, IR groups showed apparent enlarged renal tubule lumens. Both in the D1 and D3 IR group, epithelial cells were shed, and cell debris was cast in the renal tubule lumen (solid red arrow). Furthermore, as the period of IR-AKI progressed to day 7, the presence of renal interstitial fibrosis was observed (green arrow). However, these lesions were significantly reduced in the CF@P group. We visualized this improvement through pathological scoring, and the scoring results fully demonstrated the therapeutic effect of CF@P (**Figure 6E and Figure 6I**). Consistent with the H&E, PAS also showed the presence of exposed basement membrane in the IR group, as well as shed epithelial cells, cell debris, and casts. However, the same changes were rarely observed in the CF@P treatment group (**Figure 6J**).

Studies have shown that IL-6-mediated inflammatory response contributes in part toward the generation of renal injury, and it can serve as a predictor of post-AKI fibrosis in some ways 48, 49. Reducing the expression of IL-6 will help alleviate the inflammatory response and damage of AKI and help avoid complications.

Both qPCR and IF showed that the expression of IL-6 in the CF@P group was significantly lower than in the IR-AKI group (**Figure 6H and Figure 6K**). DHE and DCFH-DA were used to label ROS in the kidney tissues. As shown in **Figure S3B-C**, the IR group in D1 exhibited intense red/green fluorescence, indicating a high level of ROS. In contrast, the CF@P group showed minimal expression of red or green fluorescence, suggesting reduced inflammation. At the same time, under the double hit of inflammation and oxidative stress, apoptosis was common in IR-AKI 50, 51. The tunel immunofluorescence results showed obvious apoptosis in the D1-IR group. And in the D1-CF@P group, few apoptotic cells were seen. It fully further proved the excellent therapeutic ability of CF@P. Meanwhile, there were significantly fewer apoptotic cells in the D3 and 7-IR groups compared with the D1-IR group, which might be related to washout and self-recovery (**Figure 6L**). In summary, all results indicated that CF@P had excellent therapeutic effects and could effectively shorten the course of the disease.

### Multi-omics Revealed that CF@P Alleviated IR-AKI by Inhibiting PDK4

Advances in high-throughput technologies have provided us with new opportunities to explore and uncover the pathophysiology and drug targets of this complex disease, and these studies have generated large amounts of data and information at different molecular levels 52, 53. Herein, we employed transcriptomics and proteomics to reveal the mechanism of CF@P in treating IR-AKI.

**Figure 7:**
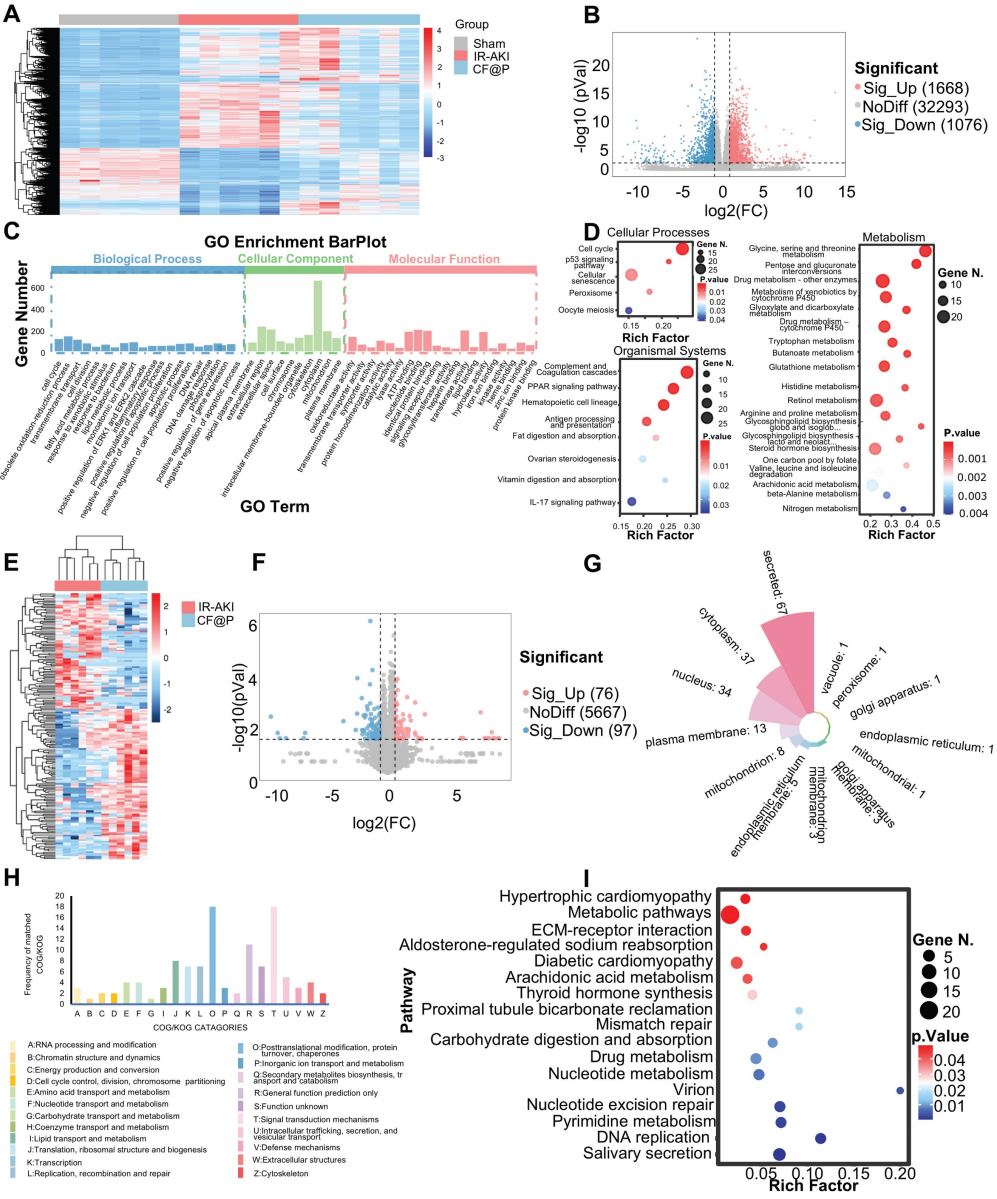
** Underlying mechanisms of CF@P on IR-AKI (D1) revealed by transcriptome and proteome. (A)** The heat map of differential genes in Sham, IR-AKI, and CF@P group. **(B)** Volcanic map of differential genes (CF@P vs IR-AKI). **(C)** GO Enrichment barplot of analysis of significantly differentially expressed genes.** (D)** Top Pathways of GO Enrichment in aspects of cellular processes, organismal systems, and metabolism.** (E)** The heat map of differential proteins in IR-AKI and CF@P group. **(F)** Volcanic map of differential proteins (CF@P vs IR-AKI). **(G)** Classifications of significantly differentially expressed proteins. **(H)** COG/KOG of significantly differentially expressed proteins. **(I)** Top Pathways of KEGG Enrichment of significantly differentially expressed proteins. N = 6.

Firstly, in transcriptomics, the heatmap showed differentially expressed genes among the three groups (**Figure 7A**). Through the setting of foldchange and *p*-value, the volcano diagram showed that compared with the IR group, 1668 genes were significantly up-regulated and 1076 genes were significantly down-regulated in the CF@P group (**Figure 7B**). Through GO enrichment analysis, genes were divided into functions: Biological Process, Cellular Component, and Molecular Function. The important GO terms in Biological Process were: cell cycle; obsolete oxidation-reduction process; fatty acid metabolic process; lipid metabolic process; inflammatory response; apoptotic process; and DNA repair. Important GO terms in Cellular Components were: intracellular membrane-bounded organelle; mitochondrion; And several important GO terms in Molecular Function: oxidoreductase activity; catalytic activity; signaling receptor binding; and protein kinase binding (**Figure 7C**). Meanwhile, KEGG enrichment analysis results showed us that in Cellular Processes, CF@P might make an effort by the cell cycle pathway; p53 signaling pathway; Cellular senescence, and so on. As for Organismal Systems, CF@P might make an effort by the complement and Coagulation cascades pathway; PPAR signaling pathway; Hematopoietic cell lineage pathway; IL-17 signaling pathway, and so on. Last, in Metabolism, CF@P might make an effort by Glycine, serine, and threonine metabolism pathway; Pentose and glucuronate interconversions pathway; Drug metabolism - other enzymes pathway; Metabolism of xenobiotics by cytochrome P450 pathway; Glyoxylate and dicarboxylate metabolism pathway and so on (**Figure 7D**). Among the proteomic characterization results, the heat map simply and directly displays the differential proteins between the two groups (**Figure 7E**). Through the setting of foldchange and p-value, the volcano diagram showed that compared with the IR group, 76 genes were significantly up-regulated and 97 genes were significantly down-regulated in the CF@P group (**Figure 7F**). **Figure 7G** showed the localization classification of these 175 significantly different proteins. The COG/KOG CATAGORIES also intuitively displayed the functions of these differential proteins through histograms, mainly focusing on: 1) O: Posttranslational modification, protein turnover, chaperones; 2) T: Signal transduction mechanisms; 3) R: General function prediction only and so on (**Figure 7H**). Through KEGG enrichment analysis at the significantly different protein levels, it was found that CF@P may exert its effect through the hypertrophic cardiomyopathy pathway; Metabolic pathways; ECM-receptor interaction pathway; Aldosterone-regulated sodium reabsorption; Diabetic cardiomyopathy; Arachidonic acid metabolism; Thyroid hormone synthesis; Proximal tubule bicarbonate reclamation; Mismatch repair; Carbohydrate digestion and absorption; Drug metabolism; Nucleotide metabolism; Virion; Nucleotide excision repair; Pyrimidine metabolism; DNA replication; Salivary secretion (**Figure 7I**).

**Figure 8:**
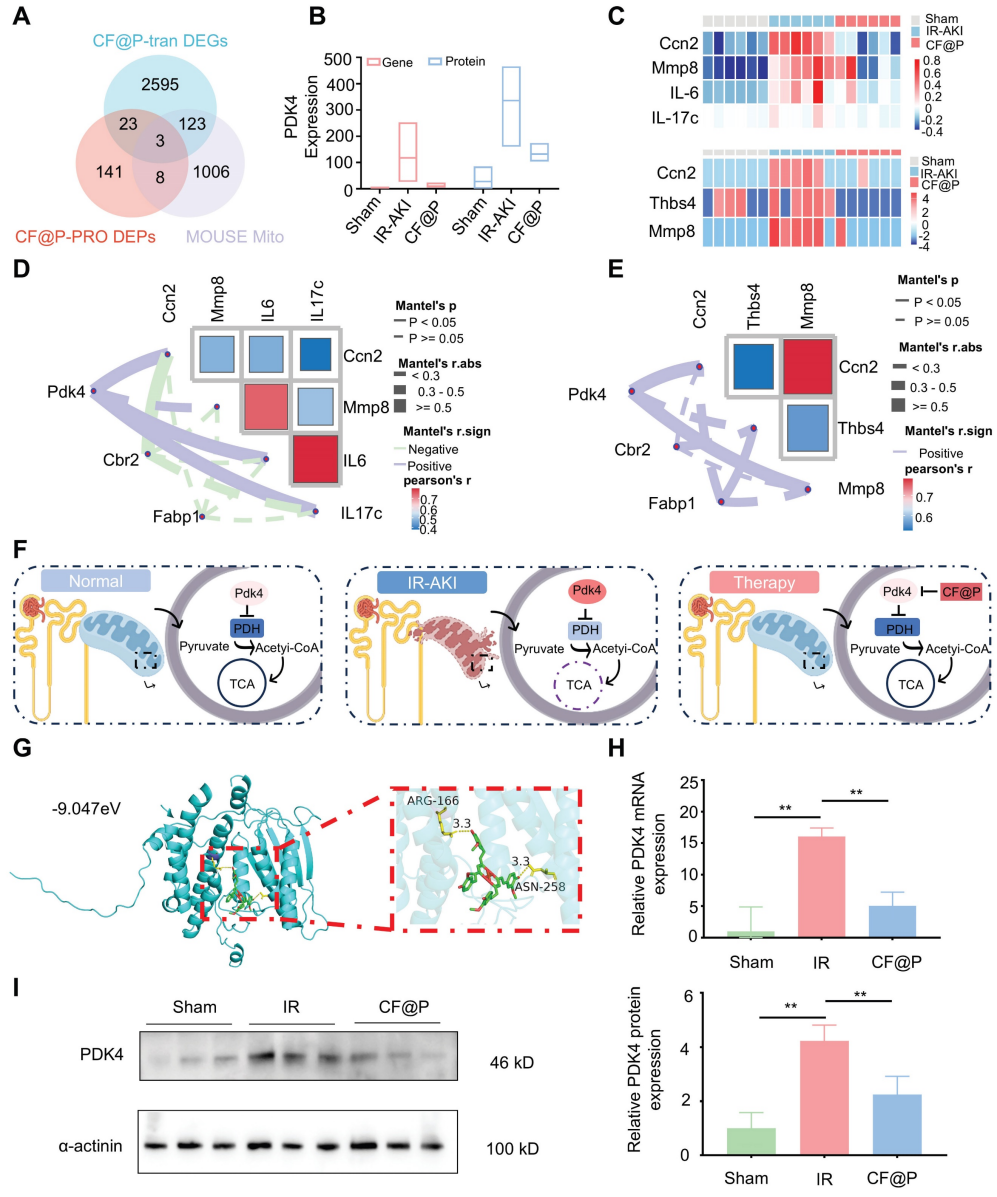
** CF@P inhibited PDK4 to attenuate IR-AKI injury revealed by transcriptome and proteome. (A)** The Venn Diagram of differentially expressed genes, proteins in CF@P and IR-AKI groups, and mice mitochondrial genes from MitoCarta3.0. **(B)** Expression of PDK4 in gene and protein of groups. **(C)** Heat map of genes and proteins related to inflammation in IR-AKI and CF@P groups.** (D-E)** Analysis of the correlation between gene and protein levels of intersection molecules and inflammation-related molecules. **(F)** Schematic diagram of the possible mechanism of CF@P in the treatment of IR-AKI. This Figure was created by Figdraw.** (G)** The molecular docking diagram and docking site magnification map of CF and Pdk4 with the lowest binding energy.** (H)** The mRNA expression levels of Pdk4 in groups (N = 3). **(I)** Kidney protein expression of PDK4 (measured using western blot analysis) and quantification (N = 3). Data are presented as mean ± SD (*P < 0.05, **P < 0.01, ***P < 0.001).

Considering the high content of mitochondria in the kidney and the excellent ROS clearance capacity CF@P, we downloaded mouse mitochondrial genes from the database MitoCarta3.0 and intersected them with differentially significant proteins and differentially significant genes. As the Venn diagram (**Figure 8A**) shown, they owned three intersections, which are: Pdk4, Cbr2, and Fabp1. In transcriptome, the expression of *Pdk4* mRNA was significantly higher in the IR-AKI group, and this trend was reversed in the CF@P group, as well as in the proteome (**Figure 8B**). Inflammation plays a central role in the occurrence and development of IR-AKI, so we selected some inflammatory genes for heat mapping (**Figure 8C**).

In transcriptome, Cellular Communication Network Factor 2 (Ccn2), Matrix metallopeptidase 8 (MMP8), IL-6, and IL-17c were selected and the expression of these inflammation-related genes was significantly decreased in the CF@P group. In proteome, Ccn2, MMP8, and Recombinant Thrombospondin 4 (Thbs4) were selected and the expression of these inflammation-related proteins was significantly decreased in the CF@P group. Further correlation analysis in transcriptome and proteome were used to explore the core target in intersections. In **Figure 8D-E**, for the selected heat maps of inflammation-related genes and proteins, the bluer the colour, the stronger the negative correlation between them; The redder the colour, the stronger the positive correlation. The solid line indicated that there was an association between the two, and the dotted line was the opposite. Meanwhile, according to Mantel's r. abs, the thicker the line, the stronger the correlation. As for the chosen inflammation-related genes and proteins, the bluer the colour, the stronger the negative correlation; The redder the colour, the stronger the positive correlation. Together, all results showed that pdk4 had a stronger association with these reduced inflammatory molecules.

Pyruvate dehydrogenase (PDH), a key regulator in mitochondrially derived metabolites, links glycolysis and the tricarboxylic acid cycle 54. This link could be destroyed by increased Pdk4 directly and cause a series of damage 55. A study strongly demonstrated that the inhibition of Pdk4 could significantly ameliorate IR-induced kidney damage by restoring the activity of PDH and then remodeling TAC cycle homeostasis 56. Consistent with this, Pdk4 also played a central role in this study. We supposed that CF@P might make an effort by inhibiting the expression of Pdk4 as **Figure 8F** showed. Further, we performed molecular docking analysis to evaluate the affinity of CF@P and Pdk4. The binding poses and interactions were obtained with Autodock Vina v.1.2.2 and binding energy was generated. Results showed that CF@P was bound to Pdk4 through visible hydrogen bonds. Moreover, the hydrophobic pockets of Pdk4 were occupied successfully by CF@P with a low binding energy of -9.047 kcal/mol, satisfying the strict criterion of binding energy less than -7 kcal/mol 57. And this result indicated highly stable binding (**Figure 8G**). Based on relative PDK4 mRNA expression (**Figure 8H** ), we further explored the protein level of PDK4 to verify our conclusion and the corresponding results (**Figure 8I**). The results determined that the renal protective effects of CF@P may be linked to the inhibition of Pdk4.

### Metabolomics Reveals the Beneficial Regulatory Effects of CF@P on Metabolites in the IR-AKI Model

**Figure 9:**
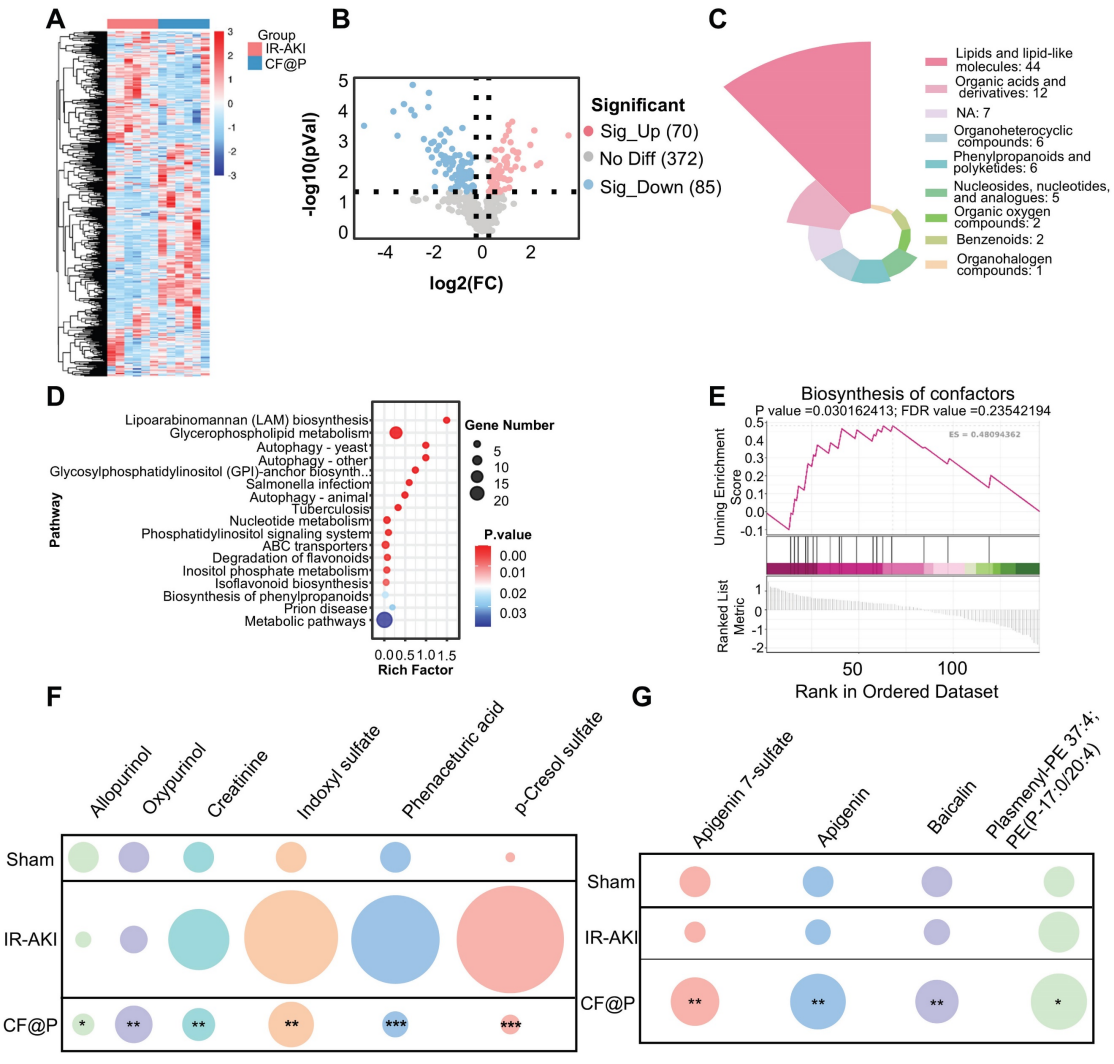
** The ability of CF@P in enhancing antioxidant substances and reducing renal toxins to alleviate IR-AKI by metabolomics. (A)** The heat map of differential metabolites in IR-AKI and CF@P group.** (B)** The Volcanic map of differential metabolites (CF@P vs IR-AKI). **(C)** MS2 Classifications of significantly differentially expressed metabolites after VIP value filtering. **(D)** Significant pathway of KEGG enrichment analysis. **(E)** Typical GSEA enrichment analysis in IR-AKI and CF@P group. **(F)** Analysis of the nephrotoxin content (compared with IR-AKI). And **(G)** Analysis of antioxidants content (compared with IR-AKI). (N = 6, *P < 0.05, **P < 0.01, ***P < 0.001).

As metabolomics provided information about biology in a specific time and place, more “proximal” to disease phenotype, we employed it to explore the effects of CF@P on metabolites in the IR-AKI model. The Heat maps of differential metabolites showed that there were a series of differences in metabolites between the IR-AKI group and the CF@P group (**Figure 9A**). Through the setting of foldchange and *p*-value, the volcano diagram showed that compared with the IR group, 70 metabolites were significantly up-regulated and 85 metabolites were significantly down-regulated in the CF@P group (**Figure 9B**). Next, we further screened by VIP values and obtained 85 significantly differentially expressed metabolites. The MS2 classification of these 85 metabolites is shown in **Figure 9C**. Among them, 44 significant differential metabolites, almost half, belonged to the category of lipids and lipid-like molecules. Meanwhile, KEGG enrichment analysis showed that CF@P may play a role through the following pathways: Lipoarabinomannan (LAM) biosynthesis; Glycerophospholipid metabolism; Autophagy; Glycosylphosphatidylinositol (GPI)-anchor biosynth...; Salmonella infection; Tuberculosis; Nucleotide metabolism; Phosphatidylinositol signaling system; ABC transporters; Degradation of flavonoids; Inositol phosphate metabolism; Isoflavonoid biosynthesis; Biosynthesis of phenylpropanoids; Prion disease; Metabolic pathways (**Figure 9D**). Further GSEA analysis showed that CF@P could affect IR-AKI metabolism by positively regulating the biosynthesis of cofactors, which plays an important role in maintaining homeostasis (**Figure 9E**). Metabolomics analysis further demonstrated a decrease in nephrotoxins (**Figure 9F**). Creatinine, indoxyl sulfate, phenaceturic acid, as well as p-cresol sulfate, were significantly reduced in CF@P-group. It effectively reduced the burden on the kidney and avoided further kidney damage. The increase of Allopurinol and Oxypurinol could reduce the level of uric acid and serve as a potent anti-inflammatory and antioxidant agent to protect IR-AKI 58. Along with this, metabolomics analysis also demonstrated a shift in other antioxidants (**Figure 9G**). The level of Apigenin 7-sulfate, Apigenin, Baicalin, and Plasmenyl-PE 37:4; PE(P-17:0/20:4) showed a significant increase trend in CF@P group. These results suggested that CF@P may reduce IR-AKI damage by enhancing antioxidant capacity.

### Biocompatibility Evaluation of CF@P

Nanozymes have emerged as a particularly promising candidate due to their excellent performance in the broad natural enzyme-mimicking capabilities, as well as their remarkable biocompatibility 59, 60. The biocompatibility of CF@P was initially investigated in both HK-2 and Raw 264.7 using the MTT assay. According to the data presented in **Figure S4A-B**, there was no notable reduction in cell viability following 24 and 48 h of co-incubation with CF@P.

Considering tail vein injection, we also co-incubated CF@P with blood cells. The results indicated that hemolysis did not occur even at the maximum concentration of 50 μg/mL, as illustrated in **Figure S4C**. We also assessed the biocompatibility in living organisms injected with 10 mg/Kg CF@P for 30 days. Additionally, **Figure S4D** demonstrated that there was no notable variation in the weight of the mice in both groups over 30 days. We assessed the biocompatibility of CF@P by conducting a histological analysis of major organs using H&E staining. The study findings indicated that the vital organs of the mice treated with CF@P did not exhibit any histological changes or damage when compared to the healthy mice (**Figure S4E**). In addition, we examined the standard blood tests and blood chemistry tests of both groups and did not identify any unusual results (**Figure S4F-O**). All these results indicated that CF@P exhibited excellent biocompatibility and could be used for animal experiments to explore its potential.

## Conclusion

In conclusion, the efficacy of CF@P in disrupting the oxidative stress-inflammation cycle during the course of IR-AKI was thoroughly demonstrated. Multi-omics analyses suggested that the renal protective effects of CF@P may be linked to the inhibition of Pdk4. Additionally, metabolic findings indicated that CF@P may increase levels of oxidants and decrease levels of nephrotoxins in the treatment of IR-AKI. The potential imaging capabilities of CF@P in T1-MRI and photoacoustic imaging have positioned it as a promising approach for AKI. CF@P, a nanomedicine with antioxidant/anti-inflammatory properties, imaging capabilities, and excellent biocompatibility, shows potential as a therapeutic approach for managing AKI in upcoming clinical settings.

## Supplementary Material

Supplementary figures and table.

## Abbreviations

ABTS: 2, 2'-azino-bis(3-ethylbenzothiazoline-6-sulfonic acid)
CAT: catalase
Cur: Curcumin
DCFH-DA: 2, 7-dichlorofluorescein diacetate
DHE: dihydroethidium
IR-AKI: Ischemia-reperfusion-induced acute kidney injury
LPS: lipopolysaccharide
Pdk4: pyruvate dehydrogenase kinase 4
ROS: reactive oxygen species
SOD: superoxide dismutase
TEM: transmission electron microscope
TMB: 3, 5, 5-tetramethylbenzidine
PDH: Pyruvate dehydrogenase
