# Supplementary material for: An enzyme-mimicking reactive oxygen species scavenger targeting oxidative stress-inflammation cycle ameliorates IR-AKI by inhibiting pyruvate dehydrogenase kinase 4
Source: Theranostics. 2024 Nov 4;14(19):7534–53. doi: 10.7150/thno.101229 (PMC11626943; doi:10.7150/thno.101229)
Supplement: Supplementary file 1 — Supplementary figures and table. [file thnov14p7534s1.pdf]

An enzyme-mimicking reactive oxygen species scavenger targeting oxidative stress-inflammation cycle ameliorates IR-AKI by inhibiting pyruvate dehydrogenase kinase 4

Supplementary Figures

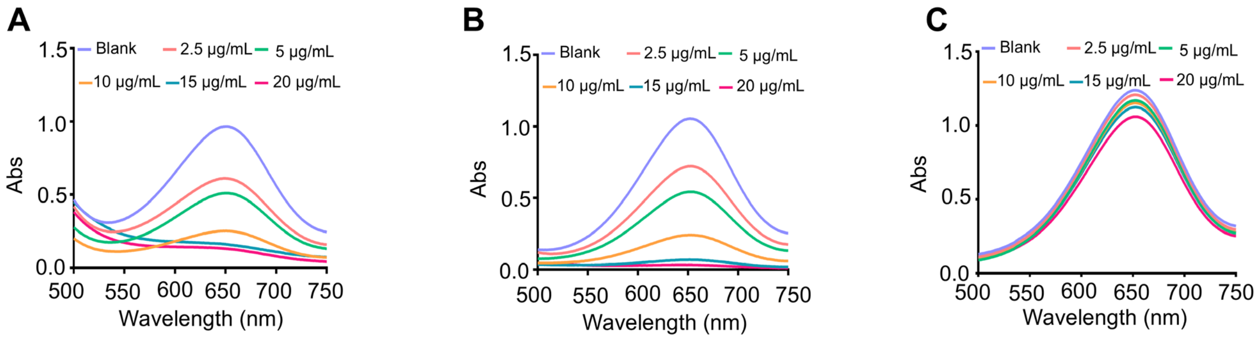

Figure S1. Supplementary characterization for antioxidant capacity of CF@P. (A-C) Detection of ox-TMB scavenging ability of CF, CF@P and PDA by Ultraviolet Spectrometer.

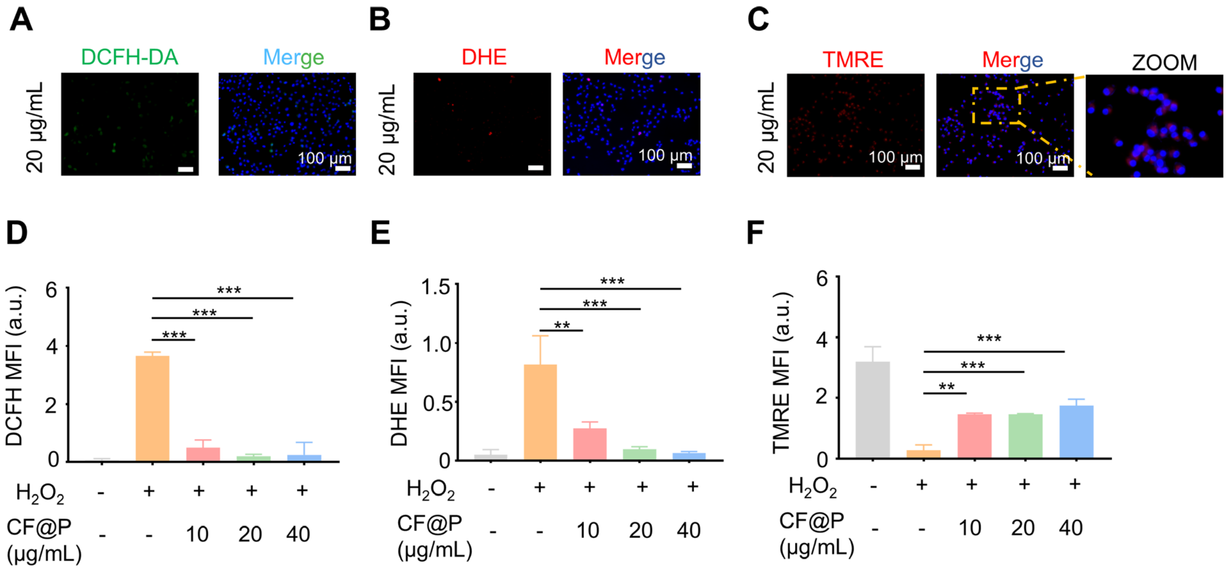

Figure S2. Supplementary materials for antioxidant detection of CF@P in HK-2 cells. (A-B) Typical DCFH-DA and DHE fluorescence images of 20 μg/mL CF@P-treated HK-2 cells (scale bar, 50 μm). (C) Typical TMRE fluorescence images of 20 μg/mL CF@P-treated HK-2 cells (scale bar, 20 μm). (D) Fluorescence quantitative analysis of DCFH in groups. (E) Fluorescence quantitative analysis of DHE in groups. (F) Fluorescence quantitative analysis of TMRE in groups.

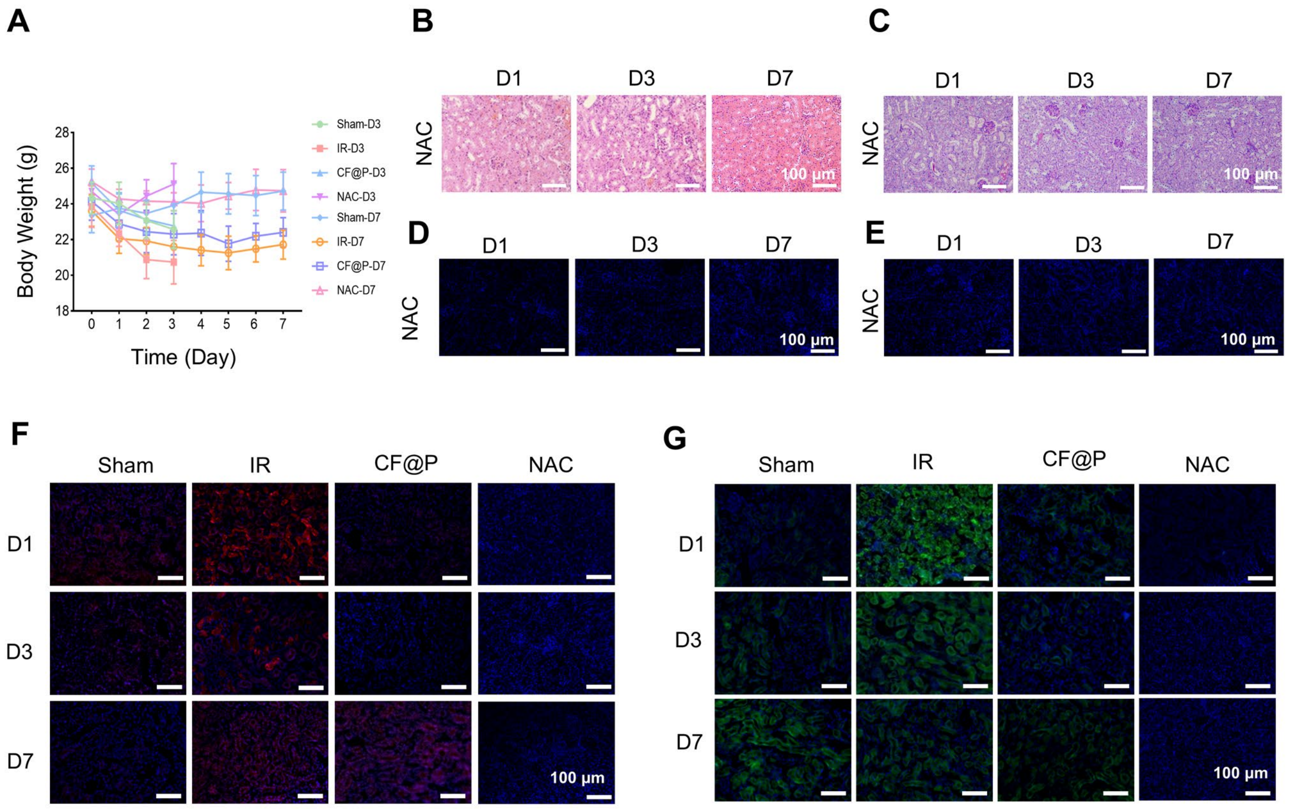

Figure S3. Supplementary materials for antioxidant detection of CF@P in IR-AKI mice. (A) Body weight changes of mice in groups. Typical H&E (B), PAS (C), immunofluorescence images of IL-6 (D) , Tunel (E) in kidney tissue of different NAC groups. Typical images of DHE (F), DCFH-DA (G) in kidney tissue of different groups (scale bar, 100 μm).

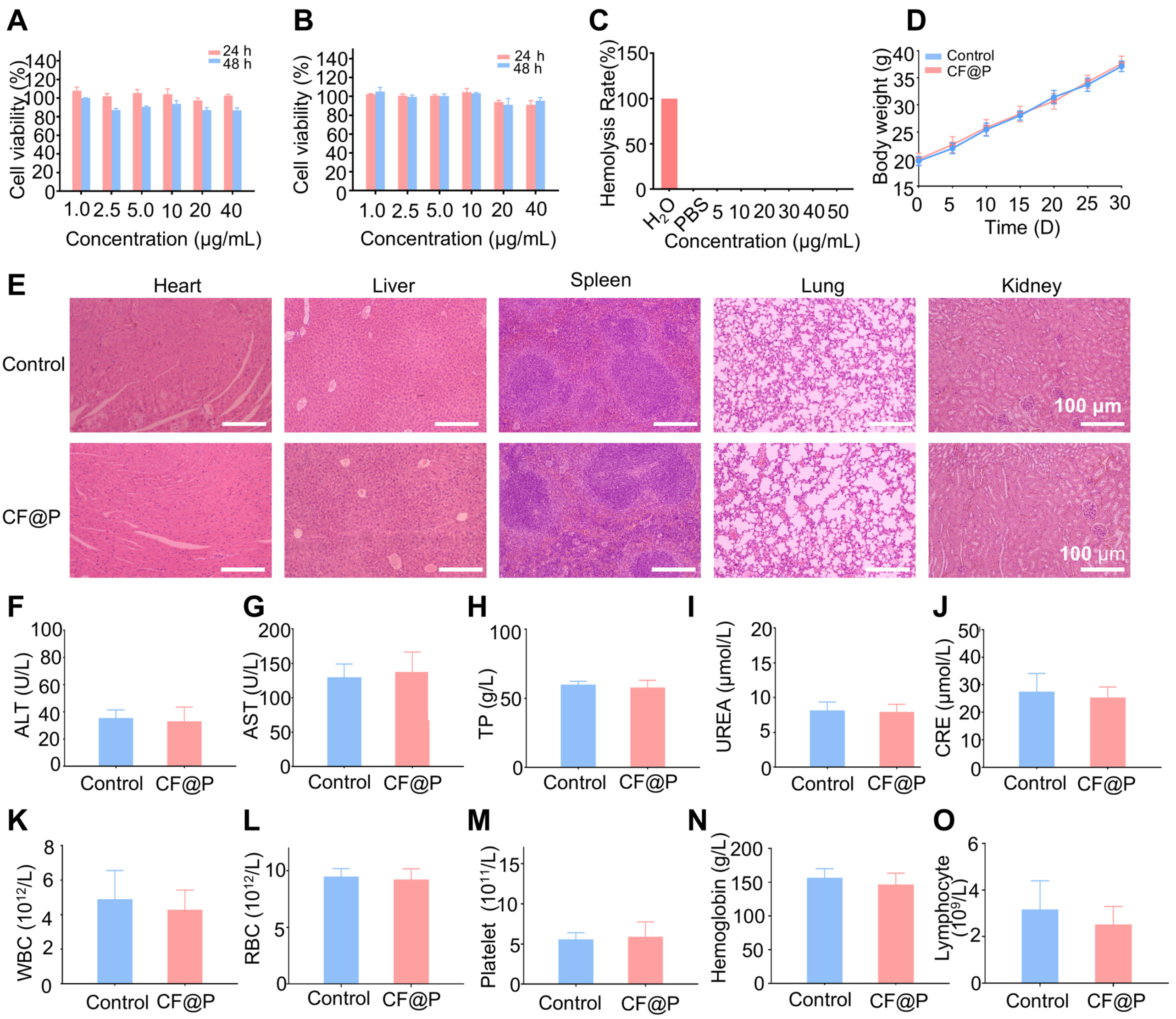

Figure S4. Biocompatibility evaluation of CF@P. (A-B) Relative viabilities of RAW 264.7 and HK-2 cells after incubation with different concentrations of CF@P (1, 2.5, 5, 10, 20, 40 μg/mL) for 24 h and 48 h. (C) Hemolysis Rate test of RCB after incubation with PBS, H_2O, and concentrations of CF@P (5, 10, 20, 30, 40, 50 μg/mL). (D) Body weight changes of mice within 30 days after injection of CF@P. (E) H&E staining for vital organs was used to assess the systemic toxicity of CF@P (scale bar, 100 μm). (F-O) Complete blood panel analysis results and blood biochemistry analysis results for treated mice. Data are presented as means ± SD (n = 6).

Supplementary Tables

Table S1. Relevant primer sequence for PCR

	F(5' to 3')	R(5' to 3')
β-actin-F(Human)	CCTGGCACCCAGCACAAT	GCCGATCCACACGGAGTACT
Kim-1(Human)	CTTCACCTCAGCCAGCAGAAA C	GCCATCTGAAGACTCTGTCACG
IL-6(Human)	GTACATCCTCGACGGCATCTC	GTGCCTCTTTGCTGCTTTCAC
β-actin(Mouse)	GAGACCTTCAACACCCCAGC	ATGTCACGCACGATTTCCC
KIM-1(Mouse)	AAACCAGAGATTCCCACACG	GTCGTGGGTCTTCCTGTAGC
NGAL(Mouse)	AGGCAGCTTTACGATGTACAGC	GAACTGGTTGTAGTCCGTGGTG
IL-6(Mouse)	ACAAGTCGGAGGCTTAATTAC ACAT	TTGCCATTGCACAACTCTTTTC
IL-10(Mouse)	GGTTGCCAAGCCTTATCGGA	CTTCTCACCCAGGGAATTCA
PDK4(Mouse)	CCATGAGAAGAGCCCAGAAGA	GAACTTTGACCAGCGTGTCTACA A
